# Mass spectrometry-guided discovery of novel GCPII inhibitor scaffolds

**DOI:** 10.3389/fphar.2025.1646207

**Published:** 2025-10-15

**Authors:** Robyn Wiseman, Ajit G. Thomas, John Janiszewski, Nate Hoxie, Chae Bin Lee, Ying Wu, Takashi Tsukamoto, Lin Ye, Michael Ronzetti, Jonathan H. Shrimp, Davina Adderley, Ganesha Rai, Rana Rais, Jesse Alt, Matthew D. Hall, Xin Hu, Stephen C. Kales, Barbara S. Slusher

**Affiliations:** ^1^ Johns Hopkins Drug Discovery, Johns Hopkins School of Medicine, Baltimore, MD, United States; ^2^ Department of Pharmacology and Molecular Sciences, Johns Hopkins School of Medicine, Baltimore, MD, United States; ^3^ National Center for Advancing Translational Sciences, National Institutes of Health, Rockville, MD, United States; ^4^ Department of Neurology, Johns Hopkins School of Medicine, Baltimore, MD, United States; ^5^ Department of Psychiatry, Johns Hopkins School of Medicine, Baltimore, MD, United States.; ^6^ Department of Neuroscience, Johns Hopkins School of Medicine, Baltimore, MD, United States; ^7^ Department of Medicine, Johns Hopkins School of Medicine, Baltimore, MD, United States; ^8^ Department of Oncology, Johns Hopkins School of Medicine, Baltimore, MD, United States; ^9^ Department of Anesthesiology and Critical Care Medicine, Johns Hopkins School of Medicine, Baltimore, MD, United States

**Keywords:** glutamate carboxypeptidase II (GCPII), screening, mass spectrometry, enzyme, inhibitor

## Abstract

**Introduction:**

There is an unmet need for therapeutics with a novel mechanism to address Q9 symptoms associated with conditions where aberrant glutamatergic neurotransmission is presumed pathogenic. One enzyme of potential relevance is glutamate carboxypeptidase II (GCPII), a brain metallopeptidase with significantly upregulated activity in nervous tissues following neurodegeneration or injury. Current inhibitors are too polar and charged leading to minimal brain penetration necessitating high systemic doses or direct brain injection. Our efforts are focused on identifying new inhibitor scaffolds with favorable brain penetration.

**Methods:**

Herein, we used a newly developed dual-stream liquid chromatography mass spectrometry (LC/MS/MS) substrate cleavage assay to screen two small molecule libraries. The two top confirmed hits were cefsulodin (IC_50_ = 2 ± 0.1 μM) and amaranth (IC_50_ = 0.3 ± 0.01 μM). The interactions of Amaranth and cefsulodin with GCPII were characterized with mode of inhibition (MOI) studies, nano differential scanning fluorimetry (DSF) thermal shift assay, and binding site was modeled with in-silico docking. As cefsulodin is an antibiotic used clinically to treat bacterial meningitis, we tested the compound’s brain pharmacokinetics (PK) in mice using a sensitive LC/MS method we developed. Moreover, following confirmation and characterization of cefsulodin and amaranth as viable hits an SAR investigation was conducted with analogs of both compounds.

**Results:**

A first derivative analysis of the DSF data revealed a shift in melting temperature of Δ 0.76 °C (±0.04) for amaranth at 25 μM and 80.41 °C (±0.05) for cefsulodin at 250 μM, suggesting both compounds are acting as stabilizers for the enzyme. Increasing concentrations of cefsulodin increased the Km of N-acetyl-aspartyl-glutamate (NAAG) as a substrate with no change in Vmax, suggesting active site competitive inhibition. In contrast, increasing concentrations of amaranth led to reductions in Vmax while the Km remained constant, suggesting a non-competitive MOI. Results from in-silico docking studies complemented this MOI data, suggesting cefsulodin likely binds in the active site while amaranth likely binds in an allosteric site. Our PK study demonstrated that administration of cefsulodin (100 mg/kg IP) led to a Cmax of 4 μM in the brain, exceeding its GCPII IC_50_ value.

**Discussion:**

Our new screening approaches identified novel inhibitors of GCPII that could serve as molecular templates for further structural optimization.

## 1 Introduction

One of the commonly shared features among a range of disorders that impact the peripheral and central nervous systems (PNS and CNS) is the dysregulation of glutamatergic signaling. Excess glutamate leads to excitotoxicity and neuronal cell death in conditions like epilepsy and stroke, and more subtle alterations to glutamate signaling pathways are also theorized to impact memory (Benveniste et al., 1984; Rothman and Olney, 1986; OLNEY et al., 1986; GREEN et al., 2021). One enzyme that has been linked to modulation of glutamatergic signaling, that is also upregulated in disease, is glutamate carboxypeptidase II (GCPII). GCPII is a metallopeptidase that cleaves the abundant neuropeptide N-acetylaspartyl glutamate (NAAG) into N-acetylaspartate (NAA) and glutamate. Upregulation of GCPII in disease means further increasing the pool of excitotoxic glutamate and consumption of the pool of NAAG which plays a role in signal modulation by acting as an agonist at metabotropic glutamate receptor 3 (mGluR3). Due to the ubiquitous nature of glutamatergic dysregulation, it is perhaps unsurprising that inhibitors of GCPII have shown efficacy in a wide range of preclinical models. In multiple models, GCPII inhibitors have demonstrated neuroprotection by reducing inflammation, cell death, and injury (Feng et al., 2012; Feng et al., 2011; Zhong et al., 2005; Zhong et al., 2006; Williams et al., 2001; Bacich et al., 2005; Slusher et al., 1999; Arteaga Cabeza et al., 2021; Harada et al., 2000; Long et al., 2005), pain attenuation (Jackson et al., 2001; Majer et al., 2003; Vornov et al., 2013; Wozniak et al., 2012; Chen et al., 2002; Yamamoto et al., 2004; ADEDOYIN et al., 2010; Nonaka et al., 2017; Yamada et al., 2012; Yamamoto et al., 2008; Yamamoto et al., 2001a; Yamamoto et al., 2007; Kozikowski et al., 2004; Carpenter et al., 2003; YAMAMOTO et al., 2001a), and alleviation of cognitive deficits related to learning and memory (Feng et al., 2011; Gurkoff et al., 2013; Gao et al., 2015; Ji et al., 2023; Rahn et al., 2012; Hollinger et al., 2016; Hollinger et al., 2022; Janczura et al., 2013; Olszewski et al., 2012; Takatsu et al., 2011; Olszewski et al., 2017; Datta et al., 2021; Bathla et al., 2023; Yang et al., 2022). The use of GCPII inhibitors has been previously reviewed (Vornov et al., 2016; Wiseman et al., 2025; Vornov et al., 2020).

Our lab has been particularly interested in the application of GCPII inhibitors as a treatment for pain and cognitive deficits. GCPII inhibitors have shown potential to be efficacious in preclinical models of inflammatory and neuropathic pain (Harada et al., 2000; Long et al., 2005; Chen et al., 2002; Yamamoto et al., 2004; ADEDOYIN et al., 2010; Nonaka et al., 2017; Yamada et al., 2012; Yamamoto et al., 2008; Yamamoto et al., 2001b; Yamamoto et al., 2007; Kozikowski et al., 2004; Sah et al., 2023; Zhang et al., 2023), and diabetic and chemotherapy induced neuropathy (Zhang et al., 2006; Zhang et al., 2002; Carozzi et al., 2010; Wozniak et al., 2012; Tang et al., 2006). Treatment with GCPII inhibitors also reduces the reinstatement of drug seeking behavior and conditioned place preference in mouse models of substance use disorder (Kozela et al., 2005; Popik et al., 2003; SHIPPENBERG et al., 2000; Witkin et al., 2002; Peng et al., 2010; Xi et al., 2010a; Xi et al., 2010b). Clinically, a novel agent to ameliorate pain without the side effects and abuse potential of current medications would be valuable.

Recent data also links inhibition of GCPII to improvement of cognitive deficits in models of traumatic brain injury (Feng et al., 2011; Gurkoff et al., 2013; Gao et al., 2015; Ji et al., 2023), multiple sclerosis (Rahn et al., 2012; Hollinger et al., 2016; Hollinger et al., 2022), schizophrenia (Janczura et al., 2013; Olszewski et al., 2012; Takatsu et al., 2011), and aging (Olszewski et al., 2017; Datta et al., 2021; Bathla et al., 2023; Yang et al., 2022). One of the potential underlying mechanisms for cognitive impairment in humans is disruption of normal glutamatergic signaling (Findley et al., 2019; CHOUDHURY et al., 2012; Polli et al., 2020; DAUVERMANN et al., 2017; Amalric, 2015). Layer III of the primate dorsolateral prefrontal cortex (dlPFC) contains a tightly regulated network of glutamatergic neurons which closely communicate with a persistent firing pattern, providing a physiological basis for high-level cognitive functions such as working memory and abstract thought (AMY et al., 2020). The delay-firing of these neurons is strengthened by the same inhibitory G protein-coupled receptor (GPCR), at which NAAG is an endogenous agonist, mGluR3. The mGlu3 receptor is encoded by the GRM3 gene (AMY et al., 2020). GRM3 has been strongly validated by multiple groups as a GWAS risk gene for schizophrenia, and a reduction in GRM3-expressing dendritic spines has been observed in Alzheimer’s disease and aging (Saini et al., 2017; Morrison and Baxter, 2012; Sartorius et al., 2008; Harrison et al., 2008; Egan et al., 2004). Additionally, downregulation of glutamatergic signaling through activation of mGluR3’s has also been shown to improve chronic pain (Mazzitelli et al., 2018).

The design of specific mGlu3 receptor agonists has proven difficult due to the receptor’s significant structural homology with mGlu2 receptors. The two receptors, despite sharing a similar structure, have different localization and function within the brain (Jin et al., 2017). A mGlu2/3 dual agonist entered Phase 3 clinical trials in patients with schizophrenia and failed to show clinical utility (Stauffer et al., 2013; Adams et al., 2014). However, a *post hoc* analysis showed the lower dose did significantly improve symptom scores compared to placebo in a subset of patients (Kinon et al., 2015). Preclinical data has supported the idea that there is an inverted U in responsiveness to dual agonist drugs, and the working hypothesis is that this is caused by overactivation of presynaptic mGluR2’s at higher concentrations, offsetting the beneficial effects of postsynaptic mGluR3 activation (Jin et al., 2017). A new strategy is needed to selectively activate mGlu3 receptors (Stauffer et al., 2013). As NAAG, the substrate of GCPII, is a selective, endogenous agonist of mGluR3’s, inhibition of GCPII provides a novel mechanism to selectively activate mGluR3 without needing to design a selective agonist. While potent (pM to nM) and selective inhibitors of GCPII have been developed, they are all polar and negatively charged at physiological pH, limiting their bioavailability and blood-brain barrier penetration. Achieving efficacy has required high systemic doses or direct brain injection, so more favorable chemical scaffolds with desired CNS exposure are required for clinical development.

Recently, a novel, reproducible liquid chromatography-tandem mass spectrometry method was developed to assist with high-throughput screening (HTS) of compounds for inhibitory activity against GCPII (Hoxie et al., 2024). The assay is more sensitive than previously developed HTS assays, only requiring 1.25 nM enzyme, 1 μM NAAG, and an incubation time of 30 min. The assay produces IC_50_ values that align well with an orthogonal, well-established and highly sensitive radioactivity assay (Rojas et al., 2002), so consequently, the two methods can be paired to identify and validate hits.

Herein, we report the results of our screening efforts with this novel LC-MS assay. To explore drug repurposing candidates and evaluate the performance of the assay, we screened the NCATS Pharmaceutical Collection (NPC; https://pubmed.ncbi.nlm.nih.gov/21525397/), an annotated collection of approved drugs and investigational drug candidates and the recently created HEAL (Help End Addiction Longterm) library (https://pubmed.ncbi.nlm.nih.gov/39144561/), a comprehensive library of annotated small molecules including drugs, probes, and tool compounds that act on published pain- and addiction-relevant targets.

The two top confirmed hits were cefsulodin (IC_50_ = 2 ± 0.1 µM) and amaranth (IC_50_ = 0.3 ± 0.01 µM). We further characterized both hits with a nano DSF thermal stability assay, mode of inhibition analysis, and *in silico* docking studies to determine the potential binding site of both inhibitors. As cefsulodin is an antibiotic which has been used clinically to treat central nervous system infections, we conducted a pharmacokinetic study to evaluate blood-brain barrier penetrability in mice. As a final step, we also conducted structure-activity relationship studies by screening a set of available analogs of cefsulodin and amaranth.

## 2 Materials and methods

### 2.1 Reagents

LC/MS-grade acetonitrile, water, methanol and formic acid were purchased from ThermoFisher (Waltham, MA, United States). D3-glutamate,^13^C_5_^15^N-acetylaspartylglutamate,^13^C_5_^15^N-glutamate, N-acetylaspartic acid, NAAG, and Glu, and diclofenac (internal standard) were purchased from Sigma-Aldrich (MO, United States). GCPII was purchased from Sino Biological (PA, United States). Clear, polypropylene, flat-bottom 384-well plates were purchased from Greiner Bio-One (NC, United States). NAAG and NAA [3H]G were obtained from Bachem AG (Bubendorf, Switzerland) and Perkin–Elmer (Boston, MA).

Compounds were obtained from multiple vendors. Cefsulodin sodium salt was purchased from Glixx (MA, United States); ceforanide was purchased from Prestwick Chemical (CA, United States) and MedChem Express (NJ, United States); cefotetan was purchased from Microsource (CT, United States) and ThermoFisher (MA, United States); cefonicid sodium was purchased form Microsource (CT, United States), Sigma Aldrich (MO, United States), and MedChem Express (NJ, United States); amaranth, trypan blue, and indocyanine green were purchased from Sigma Aldrich (MO, United States); sulfobromophthalein was purchased from Labotest (Germany).

### 2.2 Screening libraries

The NCATS Pharmaceutical Collection (NPC) contains 2,678 compounds approved for use by the U.S. Food and Drug Administration as of December 2022, along with a number of approved molecules from related agencies in foreign countries.

The HEAL library contains 2,816 compounds reported to modulate a variety of targets related to pain perception and was designed to exclude controlled substances to prevent opioid-dominated screening results.

### 2.3 GCPII inhibition determinations using a dual-stream liquid chromatography–tandem mass spectrometry-based method

The novel high-throughput dual-stream liquid chromatography–tandem mass spectrometry method used to screen for this study was recently published (Hoxie et al., 2024). Briefly, a 5 μL solution containing recombinant GCPII (SinoBio) in Tris–HCl (pH 7.4) with 1 mM CoCl2 was dispensed using a BioRAPTR 2.0 Flying Reagent Dispenser (Let’s Go Robotics) into 384-well assay plates (Greiner 784,201) pre-spotted with 100 nL of compound in DMSO using an Echo 655 Acoustic Liquid Handler (Beckman). Enzyme and compound were incubated for 15 min prior to the addition of 5 μL of ^13^C_5_^15^N-acetylaspartylglutamate (Aldrich). After 30 min s at room temperature, reactions were quenched using 0.2 μM D3-glutamate in LC/MS-grade acetonitrile and 0.1% formic acid. A 2-min liquid chromatography method utilizing a 2.1 × 30 mm BEH Amide HILIC column (Waters) was used to resolve glutamate from NAA and NAAG. A Sciex 6,500+ Q-Trap mass spectrometer was utilized for MS/MS quantification of released glutamate in the presence of compound. The integrated peak areas were normalized against the d3-glutamate internal standard, and then degree of inhibition was calculated using replicates containing no enzyme and enzyme with equivalent volumes of DMSO. Compounds were initially screened in a 10 μL reaction volume with 10 μM (final) compound dissolved in DMSO. Library compounds demonstrating ≥ 50% inhibition of glutamate release relative to DMSO controls were then re-plated in seven-point dose response to confirm activity and determine IC50 values. To further increase throughput and the efficiency of screening, LC/MS analysis of libraries was conducted in dual-stream mode on an LS-1 autosampler (Sound Analytics).

### 2.4 GCPII inhibition determinations using an orthogonal radioenzymatic-based assay

Radioenzymatic assay using ^3^H-NAAG was performed as previously described (Barinka et al., 2002). Briefly, a 50 μL reaction mixture containing NAA [^3^H]G (30 nM, 49 μCi/nmol) and GCPII (40 pM), in Tris-HCl (pH 7.4, 40 mM) with 1 mM CoCl_2_ was incubated at 37 °C for 25 min and stopped with 50 μL ice-cold sodium phosphate buffer (pH 7.5, 0.1 M). AG1X8 ion-exchange resin was used to separate cleaved [^3^H]glutamate, and the flowthrough was transferred to solid scintillator-coated 96-well plates (Packard) and allowed to dry. Radioactivity was measured by a Topcount NXT from Packard. Each compound was run in duplicate, and 6–8-point dose-response curves were generated.

### 2.5 Mode of inhibition studies

The mode of inhibition was also determined as previously described following the same procedure as the radioenzymatic-based IC_50_ assay but with modification of the concentrations of NAA [^3^H]G. (Barinka et al., 2002). Eight concentrations of radiolabeled NAA [^3^H]G (0.03125–4 μM, 1:2 dilution) were incubated with 40 pM GCPII and a range of inhibitor concentrations that surrounded their predetermined IC_50_ values, with the lowest values being close to the compound’s IC_50_ (Cefsulodin: 1.6–25 μM; Amaranth 0.3–2.4 µM). A Michaelis-Menten kinetics analysis was done to determine the K_m_ and V_max_ at each inhibitor concentration. A double reciprocal Lineweaver-Burke plot was created to visualize inhibition type (e.g., competitive, noncompetitive, etc.), and a secondary plot of K_m app_/V_max_ was used to determine the K_i_ values of cefsulodin and amaranth.

### 2.6 Modeling and docking of GCPII inhibitors

The crystal structures of GCPII in complex with a folyldi-gamma-L-glutamic acid (PDB code 4MCQ) (Navrátil et al., 2014), with a transition state analog of methotrexate-Glu (PDB code 3BI1), and with an inhibitor PSMA 1027 (PDB code 5O5U) were used for docking studies of identified GCPII inhibitors. Prior to docking the protein structures were processed using the Structure Preparation Module in the MOE program (www.chemcomp.com). Docking of cefsulodin and amaranth to the three protein structures was performed using the MOE Dock with the ligand-induced fit docking protocol, respectively. The binding affinity was evaluated using the GBVI/WSA score. 30 docking poses were generated from each target docking. The top-ranked binding poses were inspected, and the predicted inhibitor binding models were selected based on the consensus scores. The predicted binding complexes of cefsulodin and amaranth with GCPII were subjected to step-wise energy minimization, followed by 2-ns MD simulations and MM-GBSA binding free energy calculations using the program Flare (www.cresset-group.com).

### 2.7 Thermal shift assay

nanoDSF experiments were performed to assess the thermal stability of GCPII in the presence of various inhibitors. Protein samples were prepared at a concentration of 0.15 mg/mL in 50 mM Tris-HCl (pH 7.4), 1 mM CoCl_2_, 150 mM NaCl, and 0.01% Tween-20. Samples were incubated at 25 °C for 30 min prior to measurement with shaking at 1,000 rpm. Thermal unfolding was monitored using a Prometheus Panta nanoDSF instrument using standard capillaries (NanoTemper Technologies). The intrinsic fluorescence at 350 nm and 330 nm was recorded as a function of temperature, and the fluorescence ratio (350/330 nm) and its first derivative were used to track protein unfolding. A thermal gradient from 25 °C to 95 °C was applied at a rate of 0.5 °C per minute. The melting temperature (T_m_) was determined from the global maximum of the first derivative of the fluorescence ratio curve. Each condition was tested in at least three independent replicates, and data was analyzed using PR. ThermControl software (NanoTemper Technologies). One-way ANOVA with multiple comparisons to the DMSO vehicle control group was used to determine significant shifts from the control thermal unfolding profile.

### 2.8 Pharmacokinetic analysis

Pharmacokinetic studies in mice were approved by the Animal Care and Use Committee at Johns Hopkins University. Male C57BL/6 mice (25–30 g) were housed under a 12-h light-dark cycle with *ad libitum* access to food and water. Cefsulodin was administered intraperitoneally (IP) at a dosage of 100 mg/kg prepared freshly (5% DMSO and 95% HEPES saline v/v) with a dosing volume of 10 mL/kg. Blood and brain tissue samples were collected at 0.083–3 h post-administration (n = 3 per time point). Blood was obtained via cardiac puncture, and plasma was separated by low-speed centrifugation at 3,000 × g for 15 min and stored at −80 °C. Brain tissues were harvested, and immediately frozen in liquid nitrogen, and stored at −80 °C until analyses.

As cefsulodin is known to be unstable, plasma and brain tissue samples were prepared with the addition of 2% formic acid in water to adjust the pH to 5 (Lecaillon et al., 1982). For quantification of cefsulodin, plasma samples were diluted 10-fold with blank plasma, and a total of 40 μL of plasma was extracted using a protein precipitation method by adding methanol containing the internal standard (diclofenac, 1 μM). The mixture was vortex-mixed for 30 s and centrifuged at 14,000 rpm for 5 min at 4 °C. Brain tissues were homogenized in methanol at a 1:5 (w/v) ratio. A 50 μL aliquot of the homogenate was precipitated with methanol containing the internal standard, vortex-mixed, and centrifuged at 14,000 rpm for 5 min at 4 °C. A 150 μL aliquot of the supernatant was transferred to a polypropylene vial sealed with a Teflon cap and analyzed by liquid chromatography with tandem mass spectrometric methods (LC-MS/MS) as described below.

Cefsulodin concentrations in mouse plasma and brain were measured using a Shimadzu Nexera X2 LC-40D system (Shimadzu Corporation, Kyoto, Japan) coupled with an API 6500 triple quadrupole mass spectrometer (AB Sciex, Redwood City, CA, United States). Chromatographic separation was achieved using an Acquity UPLC BEH Amide column (2.1 × 100 mm, 1.7 μm; Waters, Milford, MA, United States) with a gradient mobile phase consisting of (A) 0.1% formic acid in water and (B) 0.1% formic acid in acetonitrile at a flow rate of 0.3 mL/min. Quantitation was performed in multiple reaction monitoring (MRM) mode using cefsulodin (533.03 > 123.03) and diclofenac as the internal standard (296.13 > 215.03) (Zhou et al., 2024; Park et al., 2024). Calibration curves were constructed over the range of 0.01–100 nmol/mL or nmol/g for plasma and brain, respectively.

Plasma concentrations (nmol/mL) and brain concentrations (nmol/g) were determined, and mean concentration-time profiles were plotted for pharmacokinetic analysis. Non-compartmental analysis was performed using the Phoenix WinNonlin software version 8.4 (Certara United States, Inc., Princeton, NJ) to calculate pharmacokinetic parameters, including the maximum concentration (C_max_), time to C_max_ (T_max_), and area under the concentration-time curve (AUC_0–t_).

## 3 Results

### 3.1 Libraries were screened for GCPII inhibition using the dual-stream liquid chromatography–tandem mass spectrometry-based method

Prior art compounds of varying potency were selected to validate use of the assay for identifying GCPII inhibitors including 2-(phosphonomethyl)pentanedioic acid (2-PMPA, IC_50_ = 200 pM), 2-(3-mercaptopropyl)pentanedioic acid (2-MPPA, IC_50_ = 90 nM), and quisqualic acid (IC_50_ = 10 µM). Once the assay was validated, NCATS Pharmaceutical Collection (NPC) and Help End Addiction Longterm (HEAL) compound libraries comprised of 5,494 compounds were selected for GCPII inhibition screening. Initially compounds were screened at 10 µM and hits exhibiting greater than 50% reduction of NAAG conversion relative to DMSO controls were then analyzed in a seven-point dose response curve. Of the 5,494 compounds screened at 10μM, 139 (2.5%) demonstrated ≥50% inhibition relative to the DMSO and no enzyme controls. These included compounds already reported in the literature, including 2-PMPA, 2-MPPA, Quisqualic Acid, Folic Acid and Methotrexate and several of its analogs. Of the 139 hits identified in the single point screen, 192 compounds, representing hits and numerous structural analogs, were re-plated in a 7 pt titration at 1:3 dilution and re-tested to generate dose response curves and calculate IC50 values. The most potent, confirmed hits included Cefsulodin and Amaranth, and several of their analogs (Table 1).

**TABLE 1:** Screening results of cefsulodin and amaranth using dual-stream liquid chromatography–tandem mass spectrometry-based method.

Compound	AC50 (μM)	Max resp (100uM)	HTS max resp	Structure
2-PMPA	0.030	−98.19	−100.00	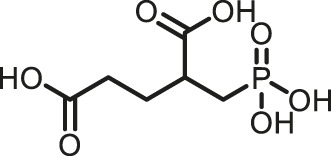
2-MPPA	2.5	−96.75	−100.00	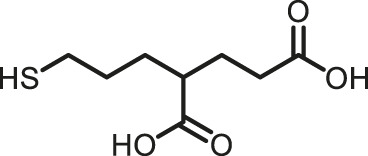
L-Quisqualic acid	7.9	−91.35	−93.07	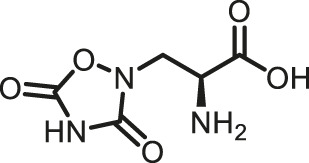
Cefsulodin	3.9	−98.19	−92.18	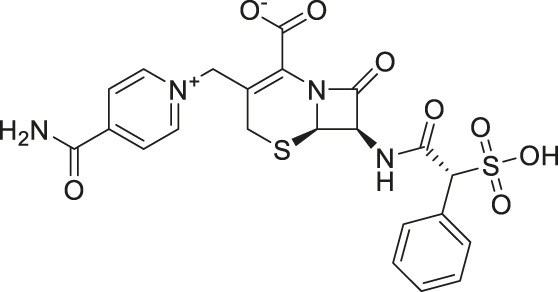
Amaranth	2.5	−101.68	−101.93	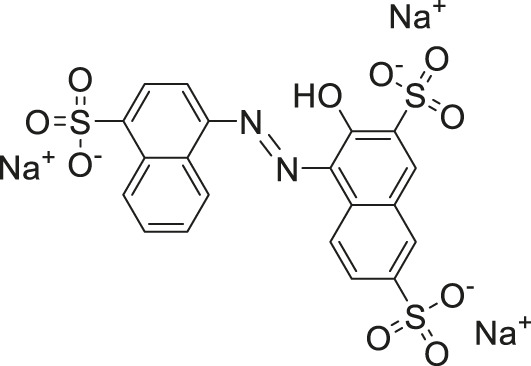

### 3.2 GCPII inhibitor hits were confirmed in an orthogonal radioenzymatic-based assay

**FIGURE 1:**
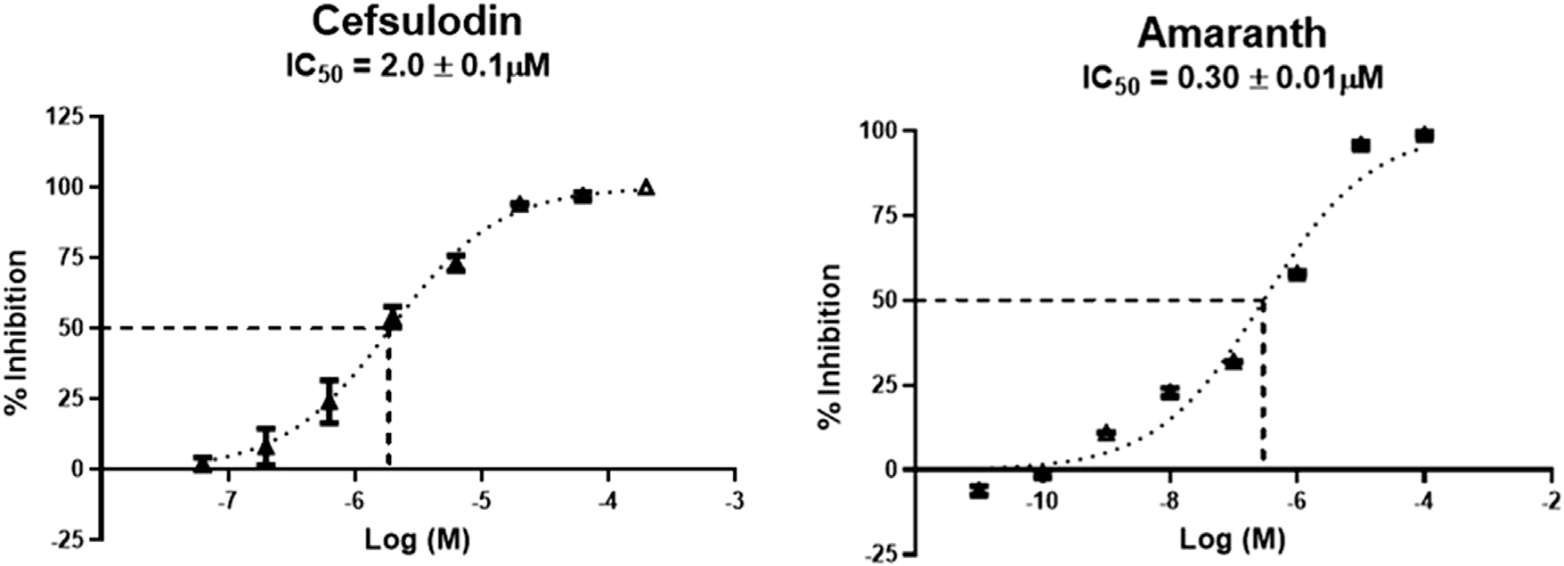
IC_50_ curves demonstrating the inhibitory potency of amaranth and cefsulodin against human rGCPII.

Hits above were next evaluated in an orthogonal radiometric assay (Rojas et al., 2002). Cefsulodin and amaranth were shown to be dose-dependent inhibitors in this assay, with IC_50_ values of 2.0 ± 0.1 and 0.30 ± 0.01 µM, respectively (Figure 1).

### 3.3 Biophysical interrogation of inhibitor binding using thermal shift assays

**FIGURE 2:**
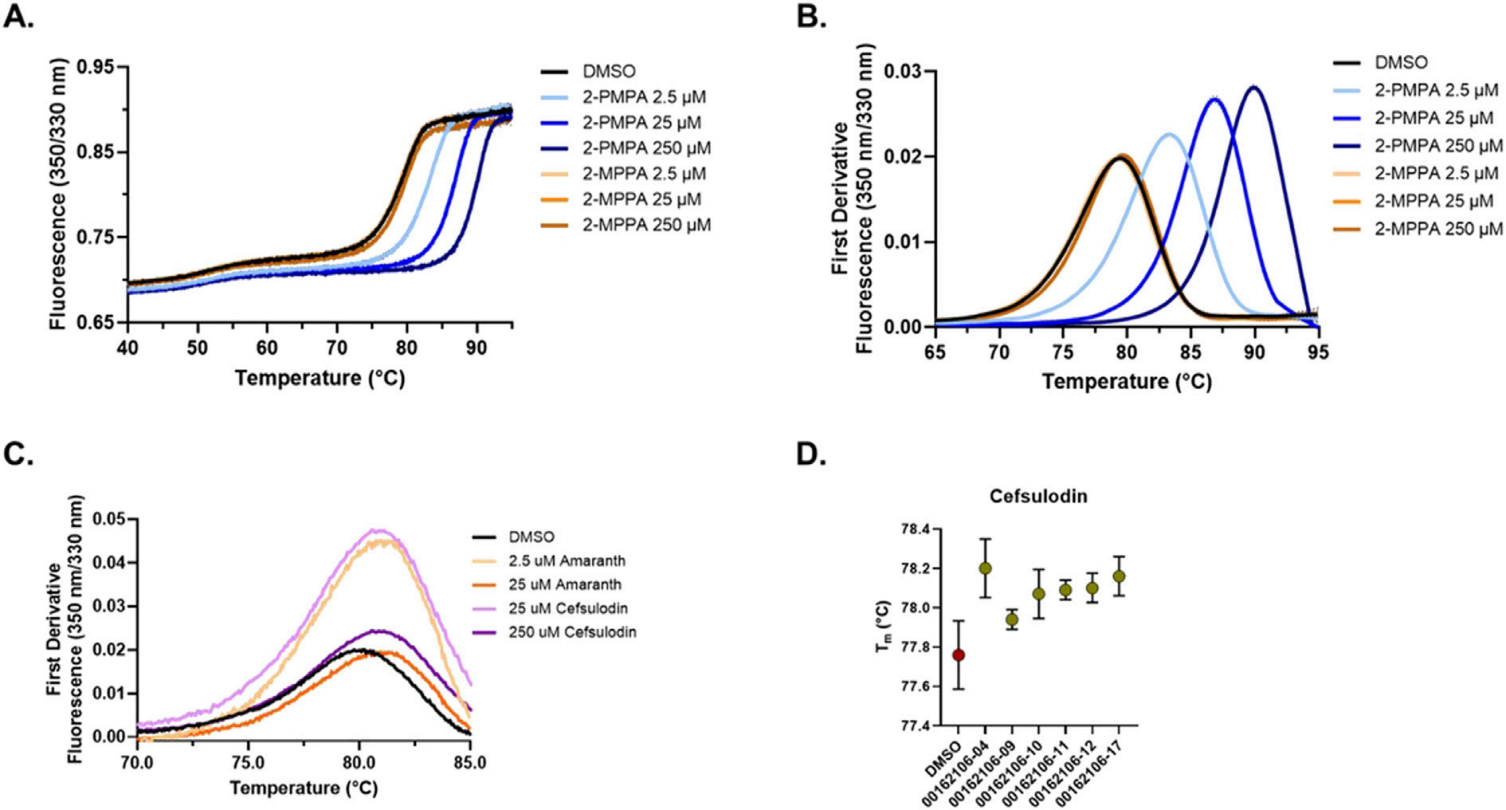
nanoDSF thermal shift testing of GCPII in the presence of inhibitors. **(A)** Thermal unfolding curves showing the ratiometric measurement of GCPII at 350 nm and 330 nm in the presence of varying concentrations of 2-PMPA and 2-MPPA. Curves shown are the average of four replicates with the 95% confidence interval shown as error dots. **(B)** First derivative analysis of nanoDSF data showing the thermal stability of GCPII with prior art molecules (2-PMPA and 2-MPPA). **(C)** First derivative nanoDSF analysis of GCPII in the presence of amaranth and cefsulodin. Curves shown are the average of four replicates with the 95% confidence interval shown as error dots. **(D)** Melting temperature (T_m_) of GCPII in the presence of cefsulodin analogs across different batches. Data shown is the average of four replicates with error bars signifying the 95% confidence interval.

Given their potency and novelty as GCPII inhibitors, we further characterized the interaction of cefsulodin and amaranth with recombinant human (rh) GCPII using a nanoDSF thermal shift assay that monitors protein unfolding through intrinsic protein fluorescence. To assess the thermal stability of GCPII in the presence of known inhibitors, experiments were conducted with increasing concentrations of 2-PMPA and 2-MPPA (Figures 2A,B). The thermal unfolding profiles showed a dose-dependent stabilization effect when compared to the DMSO vehicle control group, with increasing concentrations of both compounds resulting in a rightward shift of the unfolding transition. First derivative analysis of the fluorescence ratio (350/330 nm) revealed a clear increase in melting temperature, validating the nanoDSF thermal shift assay as a means of identifying binding to GCPII through thermal stabilization events. The T_m_ of GCPII in the presence of 2-PMPA increased from the DMSO T_m_ of 78.93 °C (±0.07) to 90.70 °C (±0.01) at 250 μM, while 2-MPPA at 250 µM led to a T_m_ shift to 79.25 °C (±0.04).

We next explored the thermal stability of GCPII in the presence of amaranth and cefsulodin (Figure 2C). Both compounds induced shifts in the unfolding transition. First derivative analysis revealed a shift in T_m_ of Δ 0.56 °C (±0.05) and 0.76 °C (±0.04) for amaranth at 2.5 and 25 μM and Δ 0.65 (±0.07) and 1.01 (±0.05) for cefsulodin at 25 and 250 μM, indicating stabilization of GCPII. Repeated testing of cefsulodin demonstrated consistent stabilization across multiple batches (Figure 2D), with minor variations between them.

### 3.4 Mode of inhibition

**FIGURE 3:**
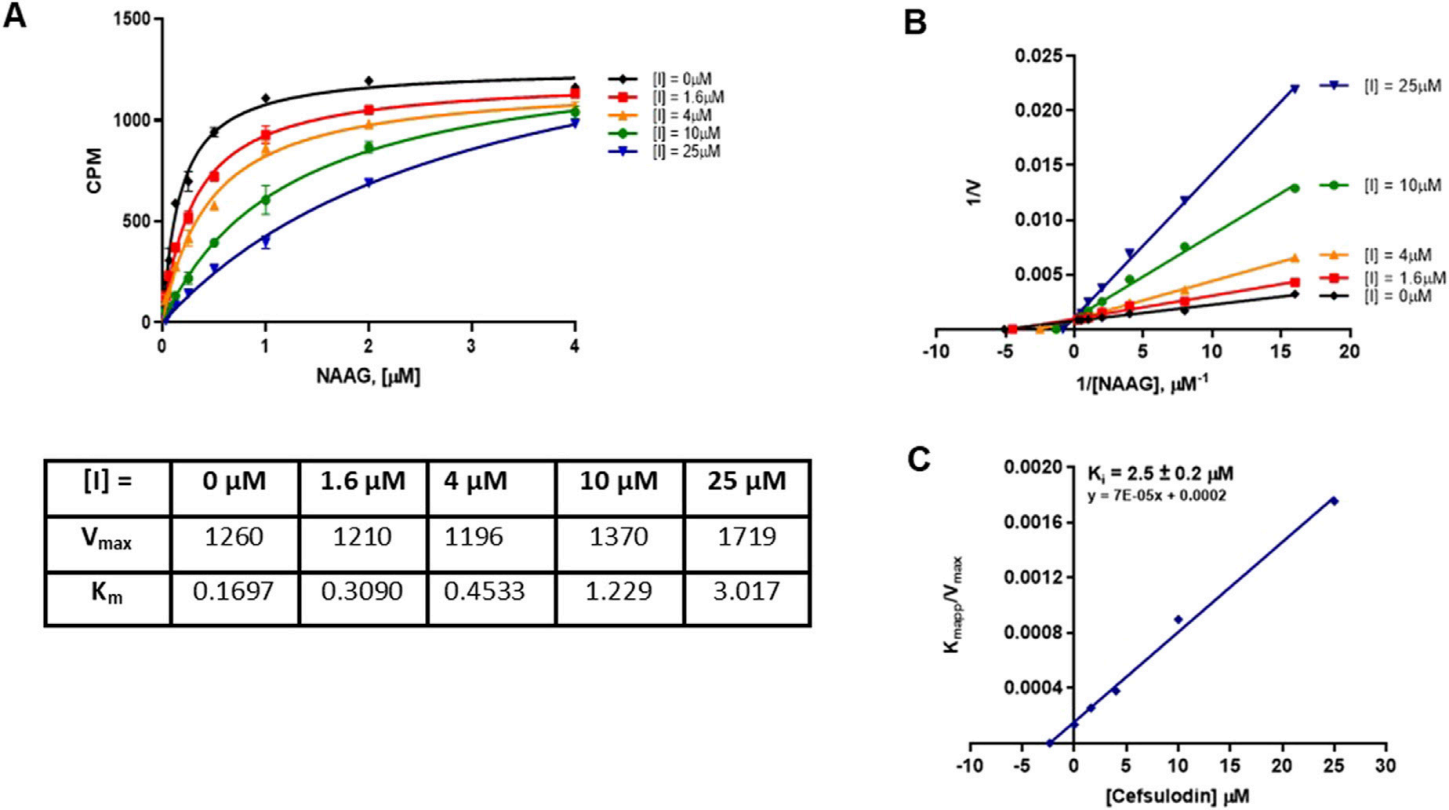
Mode of inhibition determination of cefsulodin. **(A)** Michaelis Menten curve of 40pM GCPII enzyme with varying concentrations of NAAG substrate and cefsulodin with the corresponding Km and Vmax values. **(B)** Lineweaver-Burke plot demonstrating competitive inhibition of cefsulodin. **(C)** Secondary plot (K_M__app/Vmax_ vs. [Cefsulodin]) to obtain the binding constant (K_i_ = −X intercept).

**FIGURE 4:**
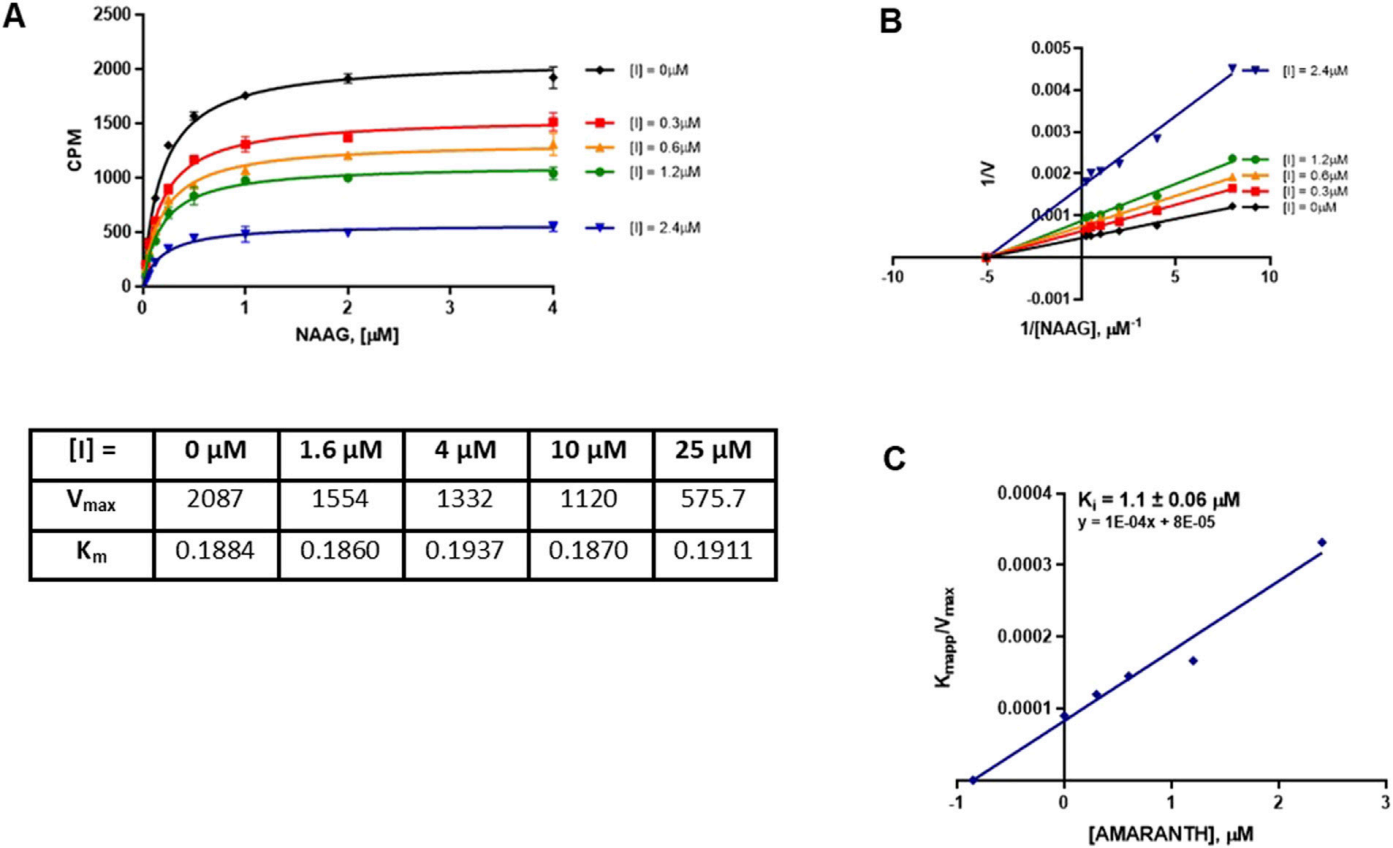
Mode of inhibition determination of amaranth. **(A)** Michaelis menten curve of 40pM GCPII enzyme with varying concentrations of NAAG substrate and amaranth with the corresponding Km and Vmax values. **(B)** Lineweaver-Burke plot demonstrating non-competitive inhibition of amaranth. **(C)** Secondary plot (K_M app/Vmax_ vs. [amaranth]) to obtain the binding constant (K_i_ = −X intercept).

We next characterized the mode of inhibition (MOI) of cefsulodin and amaranth using the radiometric assay with varying concentrations of the NAA [^3^H]G substrate to complete a Michaelis-Menten analysis. The MOI analysis of cefsulodin (Figure 3) showed that increasing concentrations of cefsulodin increased the GCPII K_m_ with NAAG as a substrate with no change in V_max,_ consistent with competitive inhibition. In contrast, increasing concentrations of amaranth led to reductions in V_max_ while the K_m_ remained constant (Figure 4), suggesting non-competitive inhibition.

### 3.5 In silico docking studies

**FIGURE 5:**
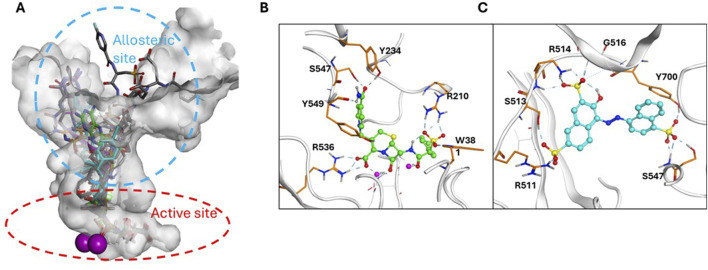
Binding model of Cefsulodin and amaranth to GCPII. **(A)** Known GCPII inhibitors bound in the active and allosteric site of GCPII. **(B)** predicted binding model of cefsulodin in the active site; **(C)** predicted binding model of amaranth in the allosteric site.

To complement the MOI studies, *in silico* docking/modeling studies were performed to determine the potential binding site of cefsulodin and amaranth within the GCPII enzyme structure. Figure 5A shows the crystal structure of GCPII overlaid with various Glu-based inhibitors. The active site is located at the bottom of the substrate binding pocket, with two Zn^2+^ ions coordinated by the side chains of His377, Asp 387, Glu425, and His553. The entrance of substrate binding cavity is extended to the apical domain and surrounded by a number of polar residues such as Arg463, Arg511, Arg 514 which are crucial to substrate recognition. Docking studies showed that cefsulodin fit well in the active site by forming metal chelation with the Zn^2+^ ions and H-bonding with residues Arg210, Arg 536, Tyr234 in the pocket (Figure 5B). Amaranth did not fit in the active site, due to its bulky structure, and is instead predicted to bind to the allosteric site at the entrance by forming extensive H-bonding interactions with residues Arg511, Arg514, Ser547, and Tyr700, likely playing an inhibitory role by blocking substrate binding (Figure 5C). The calculated total ΔG by MM-GBSA showed that the electrostatic binding energy played a major role in inhibitor binding, which is consistent with the binding model analysis (Supplementary Tables S1, S2).

### 3.6 Brain penetration of cefsulodin

**FIGURE 6:**
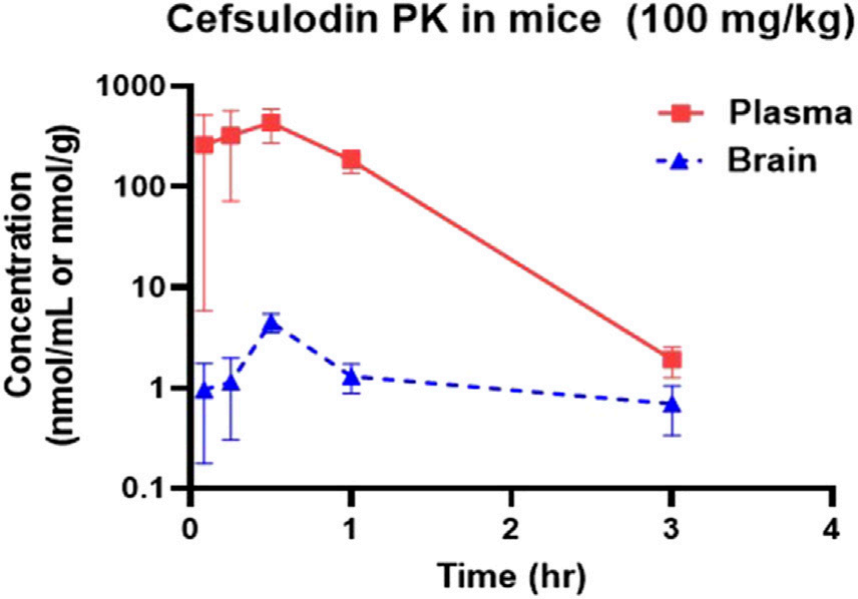
Plasma and brain concentration profiles following IP administration of 100 mg/kg cefsulodin in C57BL/6J mice.

Cefsulodin has been reported in literature to penetrate the blood brain barrier in rat models of meningitis (Meulemans et al., 2009; Meulemans et al., 1986). To confirm the brain permeation of cefsulodin when no disruption is present, we conducted a pharmacokinetic and brain penetration study in mice. The pharmacokinetic profiles of cefsulodin in plasma and brain following IP administration (100 mg/kg) are illustrated in Figure 6. Plasma levels were initially high but declined rapidly by 3 h. In contrast, brain levels were steady for all the sampled time points, reaching a maximum concentration (Cmax) of 4.9 μM at 30 min post-dose. The brain exposure measured by area under the curve (AUC) was 4.36 ± 1.12 nmol/g*h. Although brain penetration index was low, micromolar concentrations of cefsulodin were achieved in the brain, which is in line with the compound’s K_i_ value (2 µM).

### 3.7 SAR of cefsulodin and amaranth

Given the promising initial characterization data, several analogs of cefsulodin and amaranth were evaluated to obtain SAR data (Table 2). For analogs of cefsulodin, we tested cephalosporin-based compounds containing a 7-aminocephalosporanic acid (ACA) as the core scaffold, including cefonicid, ceforanide, and cefotetan. The lower inhibitory potency displayed by cefonicid and ceforanide may suggest that acidic moieties attached to the 7-amino group play a more important role in interacting with the enzyme compared to those attached to the three-position. We also tested additional sulfonic acid dyes as analogs of amaranth. Trypan blue and sulfobromophthalein were found to be equally potent as amaranth while substantial loss of inhibitory potency was observed with indocyanine green in both assays, suggesting the preference of the enzyme for arylsulfonic acids over aliphatic sulfonic acids.

**TABLE 2:** Follow up screening results of cefsulodin, amaranth, and analogs from both the dual stream LC/MS and the orthogonal radioenzymatic assay.

Compounds	IC_50__(µM)_ *(mass spec assay)*	IC_50__(µM)_ *(radioenzymatic assay)*	Structure
Cefsulodin sodium salt	3.6 ± 0.3	2.0 ± 0.1	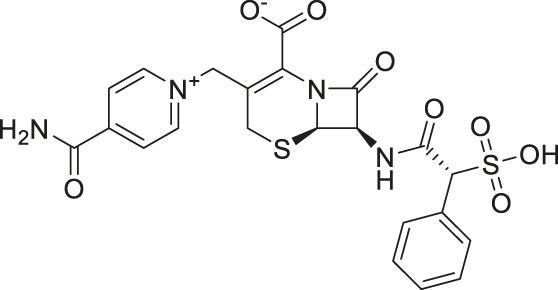
Cefonicid sodium	32 ± 2	4.9 ± 2	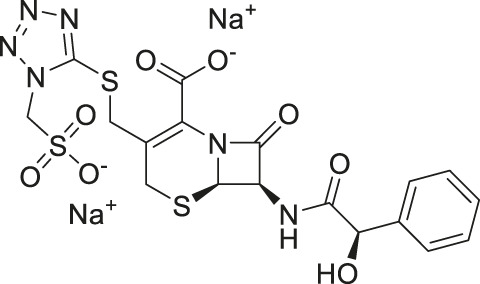
Ceforanide	29 ± 0.4	14 ± 6	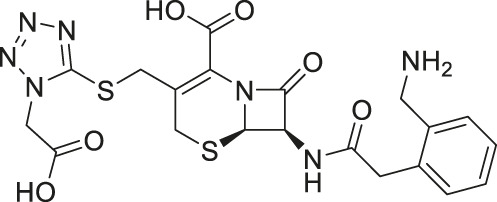
Cefotetan	9.7 ± 3	2.0 ± 1	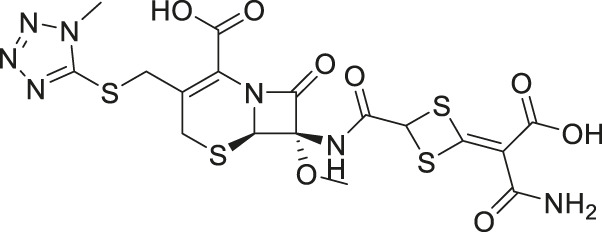
Amaranth	1.5 ± 0.3	0.30 ± 0.01	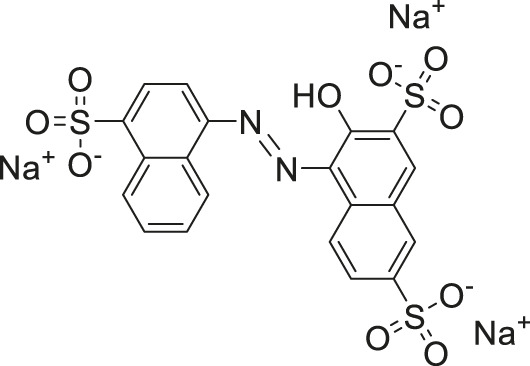
Trypan blue	2.1 ± 0.4	0.21 ± 0.05	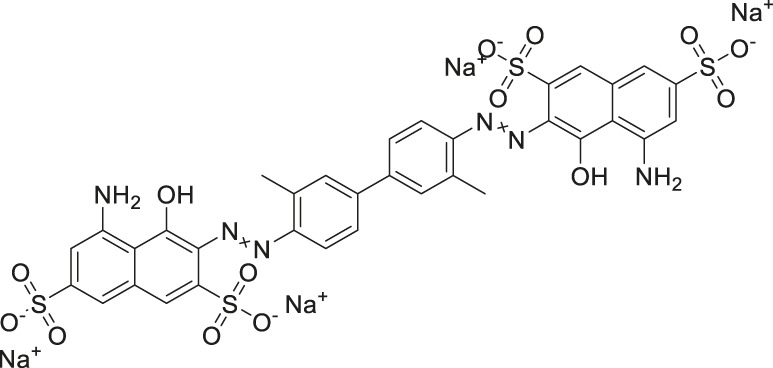
Sulfobromophthalein	0.40 ± 0.1	0.23 ± 0.1	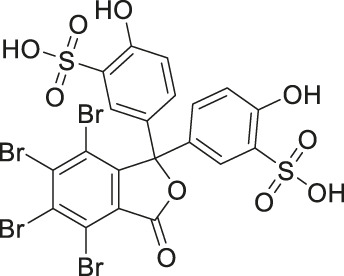
Indocyanine green	11 ± 0.2	1.9 ± 0.3	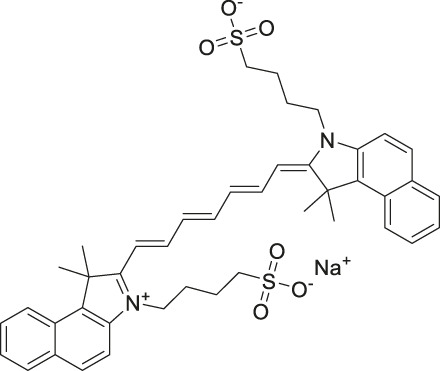

Triplicate IC_50_ values ±standard deviation.

## 4 Discussion

A novel dual-stream liquid chromatography–tandem mass spectrometry assay was utilized to identify novel inhibitors of the enzyme GCPII. For this effort, two libraries of known, small molecule drug repurposing candidates were selected. We identified the third-generation cephalosporin, cefsulodin (IC_50_ = 2.0 ± 0.1 µM), and amaranth dye (IC_50_ = 0.30 ± 0.01 µM) as inhibitors of GCPII. Several analogs of these compounds were also tested and shown to also have inhibitory activity. Direct interaction of GCPII with cefsulodin and amaranth was further supported by stabilization in the nanoDSF thermal shift assay, where an elevation in T_m_ was observed with both compounds. After validation of the potency of cefsulodin and amaranth with the MS method and an orthogonal radioactivity based enzymatic assay, we further characterized the nature of the enzyme-inhibitor interactions. We used a radioactivity-based enzymatic assay to determine the inhibition mode of cefsulodin and amaranth. For cefsulodin, V_max_ remained constant while K_m_ increased, consistent with competitive, active-site inhibition. Docking studies confirmed that cefsulodin fits within the GCPII active site, acting as a bidentate Zn^2+^ chelator, similar to known inhibitors such as 2-PMPA and 2-MPPA, but with fewer charged groups and a more hydrophobic backbone, features that may enhance drug-likeness. In contrast, amaranth reduced V_max_ without affecting K_m_, indicating non-competitive inhibition. Docking suggested that amaranth’s bulky structure cannot occupy the active site but instead binds an allosteric pocket, consistent with its distinct mechanism. Given the challenges of developing drug-like competitive inhibitors for GCPII, the potency and novel allosteric binding of amaranth make it an attractive new scaffold, especially as few allosteric GCPII inhibitors have been reported (Gori et al., 2022).

One of the driving goals of this project was to identify inhibitors of GCPII which are also able to penetrate the blood-brain barrier for the treatment of disorders involving aberrant glutamatergic transmission in the central and peripheral nervous systems such as neuropathic pain and cognitive dysfunction. Cefsulodin is an antibiotic that has been used to treat bacterial meningitis both clinically and in pre-clinical rat models of disease (Meulemans et al., 1986; Meulemans et al., 2009; Tsuchiya et al., 1978). However, little work has been done characterizing the ability of cefsulodin to penetrate the brain when profound blood-brain barrier breakdown is not present, such as in meningitis. For this reason, we selected cefsulodin for a pharmacokinetic study in mice. We found that IP administration of a 100 mg/kg dose of cefsulodin in healthy, adult mice led to brain levels above the compound’s IC_50_ for GCPII.

In conclusion, this tandem mass spectrometry method is now validated as an effective approach for identifying novel GCPII inhibitors from the two repurposing libraries. The key findings from this study are: (i) cefsulodin represents a competitive, active-site inhibitor structurally distinct from previously known GCPII inhibitors, potentially offering a novel scaffold for therapeutic development; (ii) amaranth is a potent, non-competitive inhibitor that is proposed to bind at a newly identified allosteric site, providing a new molecular scaffold for the development of allosteric GCPII inhibitors; and (iii) cefsulodin achieves brain concentrations above its GCPII IC_50_ value in healthy mice following systemic administration, suggesting its therapeutic potential in neurological disorders associated with elevated GCPII activity.

These findings also pave the way for continued efforts to discover novel GCPII inhibitors through multiple approaches. First, based on the observed SAR trends and docking analyses, future structural modifications of cefsulodin could focus on optimizing its hydrophobic backbone and fine-tuning of the chelating moiety to retain zinc coordination while reducing polar surface area, thereby enhancing CNS penetration. Second, resolving the co-crystal structure of GCPII with amaranth could enable more rational structural optimization, including the potential removal of its highly acidic moieties and a reduction in molecular weight to improve CNS permeability. Lastly, given the successful identification of two mechanistically distinct GCPII inhibitors from the two relatively focused libraries, screening larger compound libraries, especially those enriched for CNS drug-like molecules, using the dual-stream liquid chromatography–tandem mass spectrometry assay is expected to identify additional novel GCPII inhibitors with diverse mechanisms of action. These could serve as new leads for developing GCPII inhibitors with enhanced therapeutic potential in neurological disorders.

## Data Availability

The original contributions presented in the study are publicly available. This data can be found here: Wiseman, Robyn; Rais, Rana; Slusher, Barbara (2025), “CEFSULODIN LC/MS/MS DATA”, Mendeley Data, V1, doi: 10.17632/dm6xky72p5.1 https://data.mendeley.com/datasets/dm6xky72p5/1.
