# Supplementary material for: Mass spectrometry-guided discovery of novel GCPII inhibitor scaffolds
Source: Front Pharmacol. 2025 Oct 15;16:1646207. doi: 10.3389/fphar.2025.1646207 (PMC12568586; doi:10.3389/fphar.2025.1646207)
Supplement: Supplementary file 1 [file Table1.docx]

**Supplementary Table 1. H-bonding distances and angles in the cefsulodin and amaranth binding interaction with GCPII**

|  | **Residue atom** | **Distance (Å)** | **Angle** |
| --- | --- | --- | --- |
| **Cefsulodin** | R210-N1 | 2.77 | 164.1 |
|  | R210-N2 | 2.74 | 147.2 |
|  | Y234-O | 2.75 | 167.7 |
|  | W381-N | 2.91 | 175.6 |
|  | R536-N1 | 2.71 | 155.1 |
|  | R536-N2 | 3.22 | 151.2 |
|  | S547-O | 2.64 | 174.5 |
|  | Y549-O | 2.81 | 129.1 |
| **Amaranth** | R511-O | 2.78 | 171.3 |
|  | S513-O | 2.76 | 157.7 |
|  | K514-N | 3.13 | 144.7 |
|  | K514-N1 | 2.86 | 158.9 |
|  | S547-N | 2.99 | 154.5 |
|  | S547-O | 2.57 | 170.6 |
|  | Y700-O | 2.61 | 161.6 |

**Supplementary table 2. MM-GBSA calculations in the cefsulodin and amaranth binding interaction with GCPII**

| **MM-GBSA (kcal/mol)** | **Cefsulodin** | **Amaranth** |
| --- | --- | --- |
| **dG** | -212.15 | -21.01 |
| **VDWAALS** | -54.2 | -59.37 |
| **EEL** | -578.24 | -484.32 |
| **EGB** | 428.94 | 530.76 |
| **ESURF** | -8.65 | -8.08 |
